# Effect of estradiol and bisphenol A on human hepatoblastoma cell viability and telomerase activity

**DOI:** 10.1590/1414-431X20154400

**Published:** 2015-09-18

**Authors:** B.L. Xu, Q.Z. Zhao, X.Y. Gao, G.J. Hou

**Affiliations:** 1 Children’s Hospital of Zhengzhou, Zhengzhou, China; 2 Basic Medical College, Zhengzhou University, Zhengzhou, China

**Keywords:** Estradiol, Bisphenol A, HepG2, Telomerase activity

## Abstract

Sex hormones from environmental and physiological sources might play a major role in the pathogenesis of hepatoblastoma in children. This study investigated the effects of estradiol and bisphenol A on the proliferation and telomerase activity of human hepatoblastoma HepG2 cells. The cells were divided into 6 treatment groups: control, bisphenol A, estradiol, anti-estrogen ICI 182,780 (hereinafter ICI), bisphenol A+ICI, and estradiol+ICI. Cell proliferation was measured based on average absorbance using the Cell Counting-8 assay. The cell cycle distribution and apoptotic index were determined by flow cytometry. Telomerase activity was detected by polymerase chain reaction and a telomeric repeat amplification protocol assay. A higher cell density was observed in bisphenol A (P<0.01) and estradiol (P<0.05) groups compared with the control group. Cell numbers in S and G2/M phases after treatment for 48 h were higher (P<0.05), while the apoptotic index was lower (P<0.05) and telomerase activities at 48 and 72 h (P<0.05) were higher in these groups than in the control group. The cell density was also higher in bisphenol A+ICI (P<0.01) and estradiol+ICI (P<0.05) groups compared with the ICI group. Furthermore, cell numbers were increased in S and G2/M phases (P<0.05), while the apoptotic index was lower (P<0.05) and telomerase activities at 48 and 72 h were higher (P<0.05) in these groups than in the ICI group. Therefore, bisphenol A and estradiol promote HepG2 cell proliferation *in vitro* by inhibition of apoptosis and stimulation of telomerase activity via an estrogen receptor-dependent pathway.

## Introduction

Hepatoblastoma (HB) is the most common malignant, solid liver tumor in children, arising from multipotent stem cells that differentiate into liver and bile duct epithelial cells in undifferentiated embryonic tissue (1). It accounts for about 25% of pediatric liver tumors and 50-69% of malignant hepatic tumors among primary embryonal tumors (2). HB is presumed to be caused by abnormal hyperplasia and differentiation of healthy liver cells, although the details of its pathogenesis remain unknown (3).

It has been suggested that environmental factors and sex hormones play major roles in the etiology of HB (4). Estrogen maintains the function of sex organs and regulates metabolism in humans (5). Environmental sources of estrogen include pesticides, plastics, detergents, combustion products, as well as industrial and agricultural waste products (6). Once estrogen enters the body, it can affect the endocrine system and promote the growth of hormone-sensitive tumors. Correlations between estrogen and tumorigenesis have been investigated in various studies (7-10). It has been demonstrated that estrogen regulates the expression of specific biomarkers in breast cancer (11). Another study has suggested that estrogen promotes angiogenesis and, consequently, the proliferation of hemangiomas in children (12). However, there is little known about the role of estrogen in hepatoblastoma.

Telomerase is a type of reverse transcriptase consisting of a ribonucleoprotein complex with a RNA template and various catalytic and regulatory subunits. It is expressed in 98% of immortalized cell lines and >90% of malignant tumors (13). Telomerase activity is principally responsible for the infinite proliferative capacity of tumors (14).

This study investigated the effects of physiological and environmental estrogen on HB by treating human hepatoblastoma HepG2 cells with 17β-estradiol (E2) and bisphenol A (BPA), and then evaluating cell proliferation, apoptosis, and telomerase activity. The mechanism of action of these hormones was examined using anti-estrogen ICI 182,780 (hereinafter ICI).

## Material and Methods

### Cells and reagents

The HepG2 cell line was provided by the Medical School of Zhengzhou University. Phenol red-free Roswell Park Memorial Institute (RPMI) 1640 medium and fetal bovine serum (FBS) were obtained from Gibco (USA). ICI was purchased from Santa Cruz Biotechnology Co., Ltd. (China). E2 was from IBL International (Germany). The cell counting kit (CCK)-8 was from Dojindo (Japan). Dimethyl sulfoxide (DMSO) was obtained from Zhengzhou Chengxiang Chemical Technology Ltd. (China). The reverse transcription kit was purchased from Invitrogen (USA). SYBR Green supermix was from Toyobo (Japan), and the Telo TAGGG Telomerase PCR enzyme-linked immunosorbent assay (ELISA) kit was from Nanjing KeyGEN Biotech Co., Ltd. (China). Penicillin and streptomycin were purchased from Beijing BioDee Biotechnology Co. Ltd (China).

### Primer and probe design

Primers and probes were synthesized by Invitrogen. The sequence of forward primer TS was 5′-AATCCGTCGAGCAGAGTT-3′, which was labeled with biotin at the 5′-end. The sequence of reverse primer CX was 5′-CCCTTACCCTTACCCTTACCCTTA-3′. The probe sequence was 5′-CCCTAACCCTAACCCTAA-3′ labeled with digoxin at the 5′-end.

### Cell culture

HepG2 cells were cultured in RPMI 1640 medium containing 10% FBS, 100 U/mL penicillin, and 100 U/mL streptomycin at 37°C with 5% CO_2_and saturated humidity. After the cells had attached to the culture dish, the medium was replaced with phenol red-free RPMI 1640 medium, and the cells were cultured for 24 h. The cells were examined daily by phase contrast microscopy.

### Reagent preparation and determination of effective doses

BPA, E2, and ICI were dissolved in DMSO and stored at −20°C. Working solutions were prepared by diluting the stock solutions in phenol red-free RPMI 1640 medium. HepG2 cells were resuspended at 1×10^6^ cells/mL and seeded in a 96-well plate with 200 μL each well. After adherence, the culture medium was removed, and cells were washed twice with phosphate-buffered saline (PBS) before BPA or E2 was added at various concentrations (0, 2×10^−5^, 2×10^−4^, 2×10^−3^, 2×10^−2^, 2×10^−1^, 2×10^0^, 2×10^1^, and 2×10^2^ μg/mL BPA; 0, 1×10^−5^, 1×10^−4^, 1×10^−3^, 1×10^−2^, 1×10^−1^, 1×10^0^, 1×10^1^, and 1×10^2^ng/mL E2). Normal liver cells were similarly treated with the various concentrations of BPA or E2. ICI was used at 1×10^−6^ M according to a previous report (15).

### Treatment groups

Cells were divided into 6 treatment groups as follows: control (DMSO only), BPA, E2, ICI, BPA+ICI, and E2+ICI. The volume of DMSO in each group was <0.1% of the total volume.

### Analysis of cell proliferation

Cells were seeded at 1×10^5^ cells/well in a 96-well plate. After adherence, the culture medium was removed, and cells were washed twice with PBS. CCK-8 solution (10 μL) was added to each well at 0, 24, 48, 72, 96, and 120 h, and the cells were cultured for an additional 3 h before the absorbance at 450 nm (A_450nm_) was determined using a microplate reader (Bio-Rad). A growth curve was generated from the measured values.

### Examination of the cell cycle distribution and apoptosis

Cells were collected at the logarithmic growth phase and seeded at 3×10^5^ cells/25 mL culture flask. After 24 h, the cells were washed twice with PBS and subjected to the various treatments. After 48 h, 1-5×10^6^ cells were collected by trypsinization and centrifuged at 12,000 *g* for 5 min at 4°C. The cells were then repeatedly washed with PBS and fixed in pre-cooled 70% alcohol at −20°C overnight. After washing with PBS, the cells were treated with RNase A (10 μL of a 20 μg/mL stock solution in 500 μL PBS) for 30 min at 37°C, followed by centrifugation at 8,000 *g* for 5min at 4°C. The cells were then incubated with 10 μL of a propidium iodide solution (50 μg/mL in 500 μL PBS) for 30 min at room temperature in the dark. Cell cycle and apoptosis analyses were carried out by flow cytometry (BD Biosciences, USA) using CellQuest software (BD Biosciences, USA). A total of 10,000 cells was used to analyze and the cell cycle distribution with FlowJo software (USA).

### Analysis of telomerase activity

A PCR-telomeric repeat amplification protocol (TRAP)-ELISA kit (16,17) was used to determine the telomerase activity of HepG2 cells according to the manufacturer’s instructions. Briefly, the cells were collected at each time point and washed twice with normal saline. A lysis solution (200 μL) was then added to dissolve the cells. After 30 min of incubation, the cells were centrifuged at 12,000 *g* for 20 min at 4°C, and the supernatant was stored at −80°C until use. Two microliters of telomerase extraction solution incubated at 65°C for 10 min was used for the negative control. The TRAP reaction was carried out in a 50 μL volume including 2× substrate buffer (25 μL), TS and CX primers (2 μL or 100 ng each) 2 U Taq enzyme (1 μL), TRAP template (1 μL), and sterile diethylpyrocarbonate water. Sterile paraffin oil (40 μL) was used to overlay the reaction solution. Telomeric repeat sequences were synthesized by telomerase at 25°C for 30 min, and then the enzyme was inactivated at 94°C for 5 min. The PCR conditions were as follows: 30 cycles of 94°C for 30 s, 50°C for 30 s, and 72°C for 90 s, followed by final extension at 72°C for 10 min. To detect telomerase activity, a polyvinyl chloride panel was coated with 1:50 biotin-streptavidin (50 μL per well) overnight at 4°C. The following day, the panel was washed four times with cleaning solution (10 mM Hepes-KOH, 1.5 mM MgCl_2_, 10 mM KCl, and 1 mM DTT). PCR product (5 μL) was mixed with 20 μL denaturation solution (0.5% NaOH), followed by incubation for 10 min at room temperature and then addition of 225 μL hybridization solution. A total of 100 μL of the mixture was transferred to the panel, and hybridization was carried out at 37°C for 3 h. After three washes with washing buffer, 100 μL peroxidase-conjugated anti-digoxin antibody was added to the panel, followed by incubation for 30 min at 37°C. After five washes with washing buffer, 100 μL substrate buffer (containing 3,3′,5,5′-tetramethylbenzidine) was added to the panel. Color development was allowed to proceed for 10-15 min. The reaction was terminated by addition of 2M sulfuric acid. Telomerase activity was determined by measuring the A_450nm_ (reference wavelength: 630 nm) on a microplate reader. Negative and positive control measurements corresponded to A_450nm_ <0.2 and A_450nm_ >1.0, respectively.

### Statistical analysis

Data are reported as means±SD. Comparisons between groups were performed by one-way analysis of variance. MATLAB software (MathWorks, USA) was used for all statistical analyses. P<0.05 was considered to be statistically significant.

## Results

### E2 and BPA stimulate HepG2 cell proliferation

**Figure 1:**
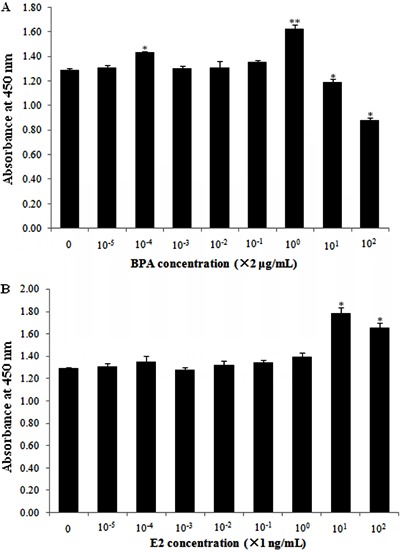
Screening for optimal concentrations of bisphenol A (BPA) and estradiol (E2) to stimulate HepG2 cell proliferation. Data are reported as means±SD. *P<0.05, **P<0.01 compared to no treatment (one-way ANOVA).

**Figure 2:**
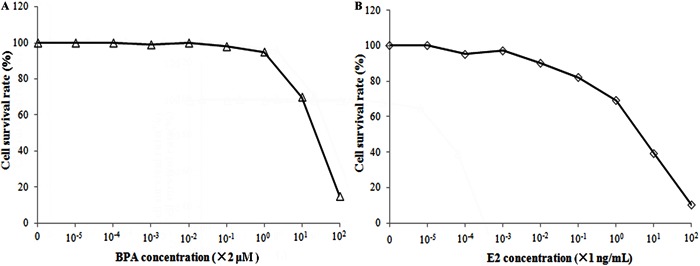
Inhibitory effect of bisphenol A (BPA) and estradiol (E2) on normal liver cell survival. The inhibitory action was stronger with increased dosage of BPA and E2.

**Figure 3:**
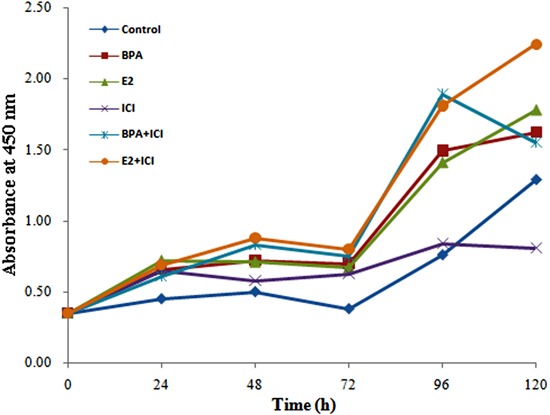
Growth curve of HepG2 cells treated with bisphenol A (BPA) or estradiol (E2) alone or in combination with the anti-estrogen ICI 182,780 (ICI).

After 120 h of treatment, the effective concentrations of E2 and BPA to stimulate HepG2 cell proliferation were 2 μg/mL and 10 ng/mL, respectively (Figure 1). For normal liver cells, both BPA and E2 had inhibitory effects on their growth (Figure 2). At various time points, BPA, E2, BPA+ICI, and E2+ICI groups had the highest proliferation rates, whereas ICI alone had little effect on cell growth (Figure 3 and Table 1). Cell numbers in the BPA group at 48, 72, and 96 h were significantly higher (P<0.01 or P<0.05) and those in the E2 group were higher at 24, 48, and 72 h (P<0.01) compared with control cells. Compared with the ICI group, cell numbers were higher in the BPA+ICI group at 48, 96, and 120 h (P<0.01 or P<0.05) and in the E2+ICI group at 48, 72, 96, and 120 h (P<0.01). These results indicate that E2 and BPA induce the proliferation of HepG2 cells.

**Table:**
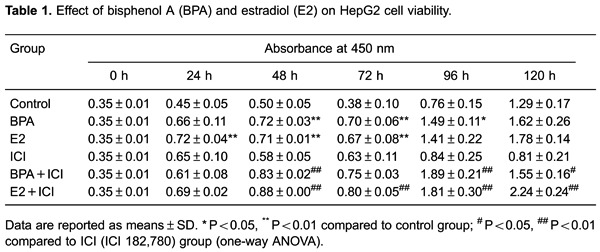

### Cell cycle regulation is affected by E2 and BPA

**Figure 4:**
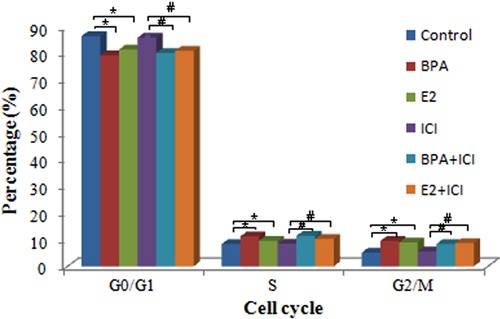
Effect of bisphenol A (BPA) and estradiol (E2) on the HepG2 cell cycle distribution. Data are reported as means±SD. ICI: ICI 182,780. *P<0.05 compared to control group; ^#^P<0.05 compared to ICI group (one-way ANOVA).

The proportions of cells in the various cell cycle phases were obviously different in E2- and BPA-treated cells and control cells (P<0.05; Figure 4). Similarly, there were significant differences in the cell cycle distributions of BPA+ICI and E2+ICI groups compared with the ICI group (P<0.05). No statistical difference was found between BPA and BPA+ICI groups or between E2 and E2+ICI groups (P>0.05). These results indicate that E2 and BPA stimulate HepG2 cell proliferation by altering the cell cycle.

### E2 and BPA inhibit apoptosis of HepG2 cells

**Figure 5:**
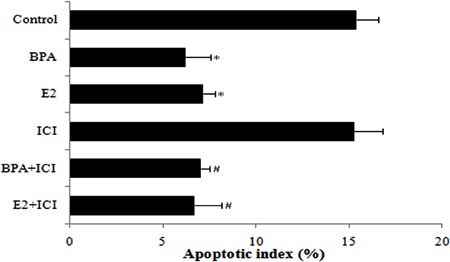
Apoptotic index of HepG2 cells treated with bisphenol A (BPA) or estradiol (E2) alone or in combination with the anti-estrogen ICI 182,780 (ICI) for 48 h. Data are reported as means±SD. *P<0.05 compared to control group; #P<0.05 compared to ICI group (one-way ANOVA).

Flow cytometry showed that the apoptotic index was markedly reduced in cells treated with E2 and BPA compared with the control group (P<0.05; Figure 5). Similarly, compared with cells treated with ICI alone, apoptosis rates were reduced in BPA+ICI and E2+ICI groups (P<0.05). Differences between control and ICI groups, BPA and BPA+ICI groups, and E2 and E2+ICI groups were not statistically significant (P>0.05). These data demonstrate that E2 and BPA inhibit apoptosis of HepG2 cells.

### Telomerase activity is induced by E2 and BPA

**Figure 6:**
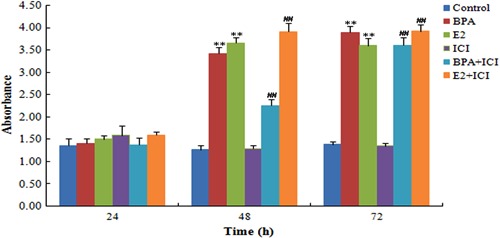
Effect of bisphenol A (BPA) and estradiol (E2) on telomerase activity in HepG2 cells. Cells were treated with BPA or E2 alone or in combination with the anti-estrogen ICI 182,780 (ICI). Telomerase activity was assessed by the PCR-telomeric repeat amplification protocol (TRAP). Data are reported as means±SD. **P<0.01 compared to control group; ^##^P<0.01 compared to ICI group (one-way ANOVA test).

Telomerase activity in HepG2 cells was evaluated by a PCR-TRAP-ELISA. Compared with the control group, telomerase activity was enhanced in BPA- and E2-treated cells at 48 and 72 h (P<0.05; Figure 6). Similarly, compared with the ICI group, telomerase activity was higher in BPA+ICI and E2+ICI groups at 48 and 72 h (P<0.05). There were no differences between ICI and control groups at any time point (P>0.05).

## Discussion

HB is the most common type of pediatric liver tumor. It is highly malignant, associated with poor outcomes, even after treatment, and characterized by occult occurrence, vascular enrichment, rapid growth, and early metastasis (18,19). The pathogenesis of HB remains unclear, but it is thought to arise from mutations and changes in hormone levels caused by environmental factors (4,20). For example, some childhood cases of HB have been linked to the use of oral contraceptives by the mother (4), suggesting the influence of estrogen. The estrogen antagonist tamoxifen has been shown to stimulate HepG2 cell activity and inhibit apoptosis (21). Furthermore, estrogen antagonists inhibit expression of the estrogen receptor and HepG2 cell proliferation *in vitro* in time- and concentration-dependent manners (22). Consistent with these previous findings, the present results showed that E2 and the environmental estrogen BPA promote HepG2 cell proliferation, which may be due to inhibition of apoptosis because treatment with these agents had no effect on the cell cycle distribution. Co-treatment with ICI did not alter the effects of E2 or BPA, indicating that these agents act via a non-estrogen receptor-dependent pathway in accordance with the known mechanism of estrogen receptor signaling (21).

The role of telomerase in tumor malignancy has been highlighted by many studies. According to a previous report, the hyperproliferation of tumor cells in 90% of human malignancies is linked to inappropriate telomerase activity (23). A study of 100 immortalized cell lines derived from 18 types of tumor tissues demonstrated that 98 cell lines had abnormally high telomerase activity by TRAP in contrast to cells from non-cancerous tissue that were negative for telomerase activity (24). Here, we showed that E2 and BPA stimulate telomerase activity in HepG2 cells. Therefore, inhibition of apoptosis by these two agents may be achieved by stimulation of telomerase activity, which suppresses telomere shortening, chromosomal damage, and ultimately apoptosis (24). This finding is substantiated by the observation that tamoxifen induces apoptosis of HepG2 cells by suppression of telomerase function (21). Taken together, these results indicate that therapeutic agents targeting telomerase may be effective for the treatment of HB. Moreover, our findings provide an insight into the mechanisms underlying the tumorigenic effects of physiological and environmental estrogens.
